# The Role of Sleep in Mediating Mental Health Symptoms During the COVID-19 Pandemic in Children with and Without ADHD

**DOI:** 10.3390/children13010082

**Published:** 2026-01-05

**Authors:** Presley MacMillan, Fakir Md Yunus, Maria A. Rogers, Yuanyuan Jiang, Emma A. Climie, Janet W. T. Mah, Penny Corkum

**Affiliations:** 1 Faculty of Medicine, Dalhousie University, Halifax, NS B3H 4R2, Canada; 2 Department of Psychology and Neuroscience, Dalhousie University, Halifax, NS B3H 4R2, Canada; 3 Psychology Department, Carleton University, Ottawa, ON K1S 5B6, Canada; 4 School of Counselling, Psychotherapy, and Spirituality, Saint Paul University, Ottawa, ON K1S 1C4, Canada; 5 Department of Educational Psychology, University of Alberta, Edmonton, AB T6G 2G5, Canada; 6 School & Applied Child Psychology, Werklund School of Education, University of Calgary, Calgary, AB T2N 1N4, Canada; 7 Department of Psychiatry, University of British Columbia, Vancouver, BC V6T 2A1, Canada; 8 Department of Psychiatry, Dalhousie University, Halifax, NS B3H 4R2, Canada

**Keywords:** pandemic, COVID-19, mental health, sleep, neurodevelopment disorder, ADHD

## Abstract

**Background:** The COVID-19 virus is a source of both acute and chronic stress for many people. This stress could uniquely impact children and their mental health. Research has shown that children with neurodevelopmental disorders such as Attention-Deficit/Hyperactivity Disorder (ADHD) are at an increased risk of negative mental health symptoms due to stress, but high-quality sleep may be associated with a protective role against these symptoms. We, therefore, aimed to investigate whether the impacts of COVID-19 and sleep problems were independently linked with children’s mental health and to examine whether sleep could mediate the relationship between COVID-19 impact and child mental health. Finally, we sought to compare the degree to which sleep problems could mediate this relationship in children without ADHD and in children with ADHD. **Methods:** In this cross-sectional study, a total of 304 parents of children were sampled from a larger study investigating the impact of the COVID-19 pandemic on Canadian families and children in the spring of 2021. Parents reported on their children’s mental health, sleep, and the impacts of COVID-19 on their child. Of the total sample, 234 children were reported as having an ADHD diagnosis, and 70 children were reported to not have ADHD. **Results:** We found that both the impact of COVID-19 and sleep problems independently and positively contributed to the mental health symptoms (*p* < 0.001) experienced by children with ADHD and without ADHD. Children with ADHD were found to have higher scores for COVID-19 child impact, sleep problems, and negative mental health. However, sleep problems had a greater impact on the mental health of children without ADHD compared to ADHD children. Additionally, the results suggest that sleep problems mediated ~20% of the relationship between COVID-19 impact and child mental health in children with ADHD and ~51% of this relationship in children without ADHD. **Conclusions:** The findings emphasize the significant role of sleep in mediating child mental health symptoms during periods of stress in children without ADHD and in children with ADHD. We highlight the importance of considering sleep quality and supporting healthy sleep in times of stress to improve child mental health symptoms.

## 1. Introduction

The COVID-19 pandemic was declared a threat to human health by the World Health Organization in March 2020 [1]. This declaration led to pandemic responses around the world that aimed to minimize the spread of COVID-19 infections. In Canada, the pandemic response involved implementing strict physical distancing requirements, closing public buildings, including schools, and limiting access to childcare, extracurricular activities, and non-essential services [2,3,4]. Overnight, people were expected to adapt to drastic changes to their everyday lives, which had implications for physical and mental health. For example, many parents of school-aged children were expected to begin working from home while simultaneously managing childcare and schooling [5,6,7]. Similarly, children were forced to switch from in-person schooling to virtual education, decreasing their social interactions and often resulting in large increases in screen time. These lifestyle changes, in addition to general uncertainty surrounding the pandemic, altered the daily routines of children, contributing to increases in sleep difficulties [8,9,10], feelings of stress [11], and negative mental health symptoms such as anxiety and depression [12,13,14] in children and adolescents [15,16].

For many during the pandemic, the fear of infection and subsequent COVID-19 restrictions became chronic stressors [17] that negatively impacted their mental health [18,19]. Chronic stress is defined as constant stress experienced over a prolonged period and has known links to many health problems, including cognitive, physical, emotional, and/or behavioural symptoms, such as changes in sleep patterns and increases in feelings of depression or anxiety [20,21,22]. Children are particularly vulnerable to the negative mental health consequences of chronic stress as a result of having less experience dealing with stressors and fewer coping strategies to employ [23]. Chronic stress experienced by children and adolescent populations can also have long-term effects that may appear later in life, such as cardiovascular disease, asthma, and depression [24,25,26]. Children with neurodevelopmental disorders such as attention-deficit/hyperactivity disorder (ADHD) are at an increased risk of experiencing mental health disorders, including anxiety and depression, when compared to children without ADHD [27,28,29]. Due to this predisposition, children with ADHD may be even more likely to experience the negative mental health impacts caused by pandemic-related stressors than children without ADHD [30,31,32]. Additionally, the pandemic disrupted many lifestyle factors that are known to be particularly important for the mental health of children with ADHD, such as consistent routines and physical activity levels [33], with emerging research showing that physical activity has a strong relationship with mental health in children [34] and adolescents [35].

Additionally, there is evidence to suggest that sleep quality plays an intrinsic role in mediating the impact of negative mental health symptoms [36,37,38,39,40,41] and that children with ADHD are at greater risk of experiencing sleep problems [42,43,44,45]. Studies have shown that during the COVID-19 pandemic, many parents perceived their child’s sleep to have worsened and attributed this poorer sleep to both increased negative mental health symptoms and behaviour changes, such as increased screen time and decreased exercise [10]. However, the degree to which sleep problems can mediate the impact of negative mental health symptoms experienced due to COVID-19 public health restrictions by children in general and children with ADHD more specifically remains unclear. Sleep has been shown to play a mediating role in the relationship between stress and mental health in adults [46,47,48]. Understanding whether sleep mediates stress and mental health in children and adolescents is important for identifying individuals at risk of poor mental health as a result of both inadequate sleep and increased stress. Similarly, recognizing whether sleep acts as a mediator in the relationship between stress and mental health could help guide interventions, such as cognitive behavioural therapy or healthy sleep practices, to reduce the negative effects of stress on mental well-being [49]. This relationship is potentially even more important for children with ADHD, as they are especially vulnerable to sleep problems such as delayed sleep onset, short sleep duration, or insomnia compared to children without ADHD [42]. Sleep problems have also been shown to worsen ADHD symptoms in children, including inattention, impulsivity, and emotional dysregulation [50]. Furthermore, the mental health conditions that children with ADHD are already at increased risk of developing, such as anxiety and depression, may be amplified by poor sleep [51] and COVID-19-related lifestyle disruptions [29].

We, therefore, examined the relationship between COVID-19 impacts and child mental health in those with ADHD and without ADHD, as measured using summed scores from a COVID-19 Child Impact Scale and the Child and Adolescent Symptom Inventory–Progress Monitor Parent Form, respectively. We further investigated whether poorer sleep (as measured using the Sleep Problems Scale for Children—Disorders of Initiating and Maintaining Sleep Index) play a role in mediating the relationship between the impact of COVID-19-related stress and lifestyle changes on children (hereafter referred to as COVID-19 child impact) and the overall mental health of children with and without ADHD. We hypothesized that (a) both COVID-19 child impact and poorer sleep have a negative impact on a child’s mental health for both groups of children and (b) that children with ADHD experience worse mental health symptoms compared with their peers. We further hypothesized that (c) poorer sleep has a strong mediating role in the relationship between COVID-19 child impact and child mental health. Moreover, we hypothesized that (d) poorer sleep has a larger mediating role for children with ADHD compared to children without ADHD.

## 2. Materials and Methods

### 2.1. Study Design, Population, and Data Collection Procedure

A cross-sectional study was conducted during the COVID-19 pandemic (May–June 2021) to capture children’s experiences of the negative consequences of COVID-19, their sleep problems, and their mental health status. This study was conducted as part of a larger study examining differences between children with and without ADHD during the COVID-19 pandemic [52]. Parents of children with or without ADHD completed self-reported close-ended structured questionnaires about themselves and completed questionnaires about their child. Inclusion criteria included that parents must have a school-aged child (kindergarten to grade 12), be residing in Canada at the time of participation, and be fluent in English to be eligible for the study. Participants were recruited for the study regardless of their location within Canada, their own biological sex, and the biological sex of their child. Furthermore, to be eligible for this study, parents were required to specify whether their child had been diagnosed with ADHD and complete survey questions pertaining to the following topics: their child’s mental health symptoms, their child’s sleep quality, and the impact of COVID-19 restrictions on their child. Of the 473 participants who provided consent, a total of 304 participants went on to complete all the questions in the study questionnaires (hosted through Qualtrics) on the topics listed above and were included in the study analysis. Of the participants who were excluded, five did not specify if their child had an ADHD diagnosis, removing them from the sample for this study. Of the remaining 164 excluded participants, most participants missed a large proportion of questions per measure (i.e., 5 or more missing questions per measure), making them unsuitable for inclusion in the study. (See Supplementary Tables S1 and S2 for a breakdown of missing response data. Supplementary Table S3 provides demographic information on the removed participants, while Supplementary Tables S4 and S5 compare the demographics of the removed and retained participants in the without ADHD and ADHD populations, respectively. See Supplementary Figure S1 for a flowchart depicting participant filtering.)

### 2.2. Study Recruitment Channels

To recruit participants, online advertisements were shared on the web pages of various Canadian national associations with ADHD as their mandate, including the Canadian ADHD Resource Alliance (CADDRA) and the Centre for ADHD Awareness Canada (CADDAC). Advertisements were also shared with research laboratories with established relationships to families of children with ADHD, and on social media platforms targeting general parenting populations. Recruited participants (n = 473) received a $20 CAD Amazon gift card as an honorarium for participating in the survey. Participants were recruited from across Canada, with the majority of participants being collected from within central Canada.

### 2.3. Data Cleaning and Preparation

#### Preliminary Data Cleaning

Online data were collected at the Rogers Child Mental Health Labs at Carleton University and the Attention, Behaviour and Cognitions (ABC) Lab at Saint Paul University (Ottawa, ON, Canada). The data were subjected to three rounds of filtering to remove data from bots. During the first round, to clean the data, all participants who met the following criteria were excluded: (1) if the survey took less than 10 min to complete; (2) if they resided outside of Canada according to the longitude and latitude given by Qualtrics; (3) if the child’s age was calculated to be younger than 3 or older than 19 years; and (4) if the survey was completed as a preview (by a member of the team).

During the second round of data filtration to identify fraudulent responses, participants were excluded if they met two or more of the following criteria: (1) if one or more participants had the same IP address, indicating they completed the survey from the same computer or tablet; (2) if one or more participants had the same email address, indicating they completed the survey and input the same email; (3) if the child’s grade did not align with their age (+/− 2 years)—for example, a 6-year-old child would be expected to be in the 1st grade, while a 3- or 9-year-old child would not be expected to be in the 1st grade; and (4) if follow-up emails were not answered.

During the third round of data filtration, participants were removed if survey emails bounced at follow-up time point T2. However, participants whose email bounced at T2 but responded to follow-up at time point 1 were still included as study participants.

### 2.4. Study Measures

#### 2.4.1. Demographics

Participant demographics were collected by asking parents to report on their own sex and gender, their child’s age and sex, their relationship to the child, their marital status, what language they speak, the highest level of education they achieved, annual household income, and Canadian province of residence at the time of participation.

#### 2.4.2. Attention-Deficit/Hyperactivity Disorder Diagnosis

Parents self-reported if their child had received an ADHD diagnosis in the past by answering a simple Yes/No question. Parents were also asked to report on who made the diagnosis of ADHD (“Who made the diagnosis? Family Physician, Paediatrician, Psychologist, Psychological Associate, Psychiatrist, Other”). Previous studies have found that parents self-reporting the mental health condition of their children is a reliable way to determine the presence of mental health conditions [53].

#### 2.4.3. The Child and Adolescent Symptom Inventory–Progress Monitor Parent Form

The Child and Adolescent Symptom Inventory–Progress Monitor Parent Form (CASI-PM-P) [54] is used to evaluate symptoms related to eight mental health disorders (ADHD—Predominantly Inattentive Presentation (ADHD-I), ADHD—Predominantly Hyperactive Presentation (ADHD-HI), Oppositional Defiant Disorder (ODD), Generalized Anxiety Disorder (GAD), Conduct Disorder (CD), Separation Anxiety Disorder (SAD), Social Phobia (SP), and Major Depressive Disorder (MDD)) listed in the *Diagnostic and Statistical Manual of Mental Disorders* (DSM) (5th edition) [55]. The CASI-PM-P includes 29 items, 28 of which measure symptoms of the above-noted disorders, with the last item asking about functional impairment related to these symptoms. The CASI-PM-P has strong internal consistency for each age group among each different mental disorder symptom category [54]. Internal consistency values in the original study ranged from [α = 0.62–0.92] across all age groups [54].

For the current study, the CASI-PM-P [α = 0.941] scale was used to capture the severity of a child’s mental health problems. Parents/caregivers were asked to indicate the severity of behavioural problems observed in their child during the previous month by answering questions such as: “Fails to give close attention to details or makes careless mistakes”, “Has difficulty remaining seated when asked to do so”, “Worries that parents will be hurt or leave home and not come back”, or “Defies or refuses what you tell him/her to do.” (A full list of questions can be found in Supplementary Table S6, and descriptive statistics for children without and with ADHD can be found in Supplementary Tables S7 and S8, respectively). Parents used a 4-point Likert scale from never (0), sometimes (1), often (2), or very often (3) to assess the frequency of each behaviour outlined in the questions. Symptoms rated by caregivers as “often” or “very often” were considered to be present. Scores across all 29 items were summed, with higher scores indicating increased symptom severity and increased overall mental health problems [54]. For this study, summed scores were used to provide a measure of cumulative mental health symptom burden [54,56], as our focus was on general mental health impacts rather than on impacts aligned with discrete diagnoses captured within the CASI-PM-P form. Response scores had a possible range from a minimum value of 0 to a maximum value of 116, with higher scores indicating worse mental health symptoms. The summed score from CASI-PM-P responses is hereafter referred to as “child mental health” for simplicity.

#### 2.4.4. COVID-19 Child Impact Scale

The COVID-19 Child Impact Scale is a modified version of the COVID-19: Supporting Parents, Adolescents and Children during Epidemics (Co-SPACE) Impact Scale [57], which was adapted for our study [α = 0.823]. The original scale, developed as part of the Co-SPACE initiative [57], was designed to better understand how families coped during the pandemic. The measure that was adapted for the current study consists of eight items regarding possible changes and consequences of the pandemic that children may have experienced (e.g., increased screen time, increased stress, decreased physical activity, less bedtime routine, fewer daytime routines and structure, increased anxiety, decreased socialization, or poorer diet). A full list of questions can be found in Supplementary Table S9. Caregivers were asked to consider the past week and rate each item based on how much it applied to their child on a scale from not at all (1), a bit (2), a lot (3), or completely (4), where higher scores indicated more significant disruption to the families because of COVID-19. Scores across all eight questions were summed to create an overall variable of the impact of COVID-19 on children (termed COVID-19 child impact), with a minimum possible score of 8 and a maximum score of 32.

#### 2.4.5. Sleep Disturbance Scale for Children—Disorders of Initiating and Maintaining Sleep Index (SDSC-DIMS)

Five items from the Sleep Problems Scale for Children (SDSC) [58] were used to evaluate symptoms related to difficulties initiating and maintaining sleep ([α = 0.710] for the current study). Symptoms related to difficulties initiating and maintaining sleep were selected for this study as issues with sleep onset and maintenance in children are associated with poorer emotional functioning and behavioral difficulties, as well as increased risk of psychiatric conditions when untreated [59,60]. In addition, these sleep problems are particularly important for children with ADHD, as children with ADHD often have higher rates of insomnia, delayed sleep phase, and disrupted sleep continuity when compared to children without ADHD [61,62,63,64].

Five items from the DIMS subscale were selected from the 26-item SDSC questionnaire that are used to evaluate specific sleep problems in children and to provide a measure of overall sleep problems. We did not investigate other sleep disorders, such as sleep breathing disorders (SBD) or sleep–wake transition disorders (SWTD), which were included in the original scale [58]. For the first item of the scale, caregivers report on how long it takes for their child to fall asleep after going to bed, rated on a scale from less than 15 min (1), 15–30 min (2), 30–45 min (3), 45–60 min (4), and more than 60 min (5). The remaining items assess how often challenging sleep behaviours (e.g., “The child goes to bed reluctantly”, “The child has difficulty getting to sleep at night (and may require a parent to be present)”, “The child wakes up two or more times a night”, and “After waking up in the night the child has difficulty falling asleep again by himself or herself”) occurred within the past month using the following scale: the behaviour never occurs (1), the behaviour occurs 1 or 2 times a month (2), the behaviour occurs 1 or 2 times a week (3), the behaviour occurs between 3 and 5 nights a week (4), and the behaviour happens every night (5). A full list of questions can be found in Supplementary Table S10. The scores of the five questions were summed to measure overall sleep problems (hereafter referred to as sleep problems), with a minimum possible score of 5 and a maximum score of 25. Using this scale, higher values indicate greater sleep problems.

#### 2.4.6. Statistical Analysis

We used a chi-square test (χ^2^) to examine the association of participants’ demographics across two study samples (i.e., ADHD and without ADHD). Independent t-tests were used to examine mean differences between the two samples for continuous variables, and the Pearson correlation (r) were used to understand the correlation among the key study variables and to further assess if there were issues of collinearity indicated by the value of the Variance Inflation Factor (VIF). VIF for each measure was less than five, suggesting that the degree of collinearity was not problematic [65].

We conducted two separate multi-level mixed-effects model analyses for children without ADHD and children with ADHD. We used multilevel modelling to estimate both unstandardized and standardized beta coefficients, capturing variability at both the measure level (within individual measures) and the group level (within the groups of measures). The analysis examined the degree to which the outcome variable (child mental health) could be estimated using the COVID-19 Child Impact and Sleep Problems variables. To obtain a better understanding of variability in the estimates, we ran four models, where Model 0 (the null model) estimated the unadjusted random effect on the outcome variable. We only included variables that showed a significant association/correlation in our univariate analyses to prevent either under- or overestimating the results [66,67].

Furthermore, we considered region as a random effect variable to account for potential clustering of participants and unobserved contextual influences, such as differences in COVID-19 restrictions. The regions are defined as Maritime provinces (N.S., N.B., N.L., and P.E.I.), Central Canada (O.N., Q.C.), Prairies and Northern Territories (A.B., M.B., S.K., N.T., N.U., and Y.K.), and West Coast (B.C). During the data collection period the restrictions varied across the country. For example, in Nova Scotia during the data collection period, indoor and outdoor gatherings were restricted to the household bubble, and all schools, all non-essential businesses, and all fitness and recreational facilities were closed [68]. In Alberta, all schools were closed, retail stores were limited to 10% capacity, outdoor gatherings were restricted to 5 people, and all outdoor sports and recreation activities were prohibited except between members of a household [69]. In Ontario, essential retail stores were limited to 25% capacity, outdoor social gatherings were allowed up to 10 people, outdoor dining was allowed up to four people (or 1 household) per table, non-essential retail stores were allowed to operate at 15% capacity, and outdoor fitness classes were allowed up to 10 people [70]. As region was not our primary variable of interest, modelling it as a random effect allowed us to control for random variability without overfitting the model. Model I and Model II are both partial models estimating the unadjusted effect of COVID-19 child impact and child sleep on the child mental health, respectively. Model III, the full model, included both predictor variables to better understand their adjusted effects. We further reported the variance of each model along with the Akaike Information Criterion (AIC) to understand the variance that occurred in the model and to identify the best model fit.

Since the mixed-effects model indicated that both predictors were significantly correlated [*p* < 0.05] with each other and had an independent, significant linear relationship with the outcome variable, mediation is warranted to better understand the direction of the relationship [71]. We carried out two separate single mediation analyses to understand if poorer child sleep mediates the relationship between COVID-19 child impact and child mental health, both for ADHD and non-ADHD samples, by using the ‘medmod’ [72] add-in for jamovi (v 2.0; The jamovi project 2024) [73]. We established 5000 bootstrapped bias-corrected 95% confidence intervals by using the standard estimation method to estimate the indirect effect in the mediation model.

## 3. Results

### 3.1. Participant Demographics and Group Comparisons

Table 1 presents participants’ demographic information in the samples without ADHD and with ADHD. The study consisted of a total of 304 participants (41.8% female; mean age = 9.78 years, SD = 2.97), of whom 234 children had a diagnosis of ADHD. We found that the mean scores of child mental health, COVID-19 child impact, and poorer child sleep were significantly higher among the ADHD children compared to the children without ADHD. The two groups did not differ on any of the demographic variables, including the child’s age and sex, parents’ sex and gender, relationship to the child, parents’ education, marital status, the family’s location in Canadian provinces, the family’s income, and the primary language spoken in the home. Pearson correlations indicated that all three key study variables were moderately and significantly correlated with each other across the two samples; however, VIF indicated no collinearity issues (VIF < 5).

Table 1 represents the descriptive statistics of the study population based on the sample. The mean differences represent raw scores obtained from participant responses to survey questions. Response ranges for neurotypical children for child mental health are min = 1 and max = 55; COVID-19 child impact min = 8 and max = 28; and sleep impact neurotypical min = 5 and max = 20. For ADHD children, child mental health are min = 7 and max = 87; COVID-19 child impact min = 8 and max = 32; and sleep impact neurotypical min = 6 and max = 23

Supplementary Table S3 contains demographic information on the study participants removed due to missing data. This sample consisted of a total of 164 children (45.1% female; mean age = 9.58 years, SD = 2.67). A significant difference was observed, with children with ADHD having higher reported child mental health problems than children without ADHD. In addition, Supplementary Table S3 showed a small but significant difference in participant geographical location. One possible explanation for these findings is that varying levels of COVID-19 restrictions across different regions of Canada may have contributed to the difference in child mental health and participant response rate.

### 3.2. The Impact of COVID-19 and Sleep Problems on the Mental Health of Children with and Without ADHD

Table 2 and Table 3 show multi-level mixed models for child mental health in samples without ADHD and with ADHD, respectively. Four different models were designed, including a null model (Model 0) that estimated the impact of random effects on the outcome variable.

In children without ADHD, we found that both COVID-19 child impact (Model I) and sleep problems (Model II) were significantly and independently linked with higher child mental health scores (i.e., more negative mental health status) [β 2.11 (1.56; 2.66) and β 3.60 (2.93; 4.27), respectively]. These estimates were slightly reduced [β 1.00 (0.48; 1.53) and β 2.72 (1.95; 3.48), respectively] but were still significantly linked when both were included in the same model (Model III). Model III, which contained both COVID-19 child impact and sleep problems variables, was found to be the best model as determined by having the lowest AIC values (497.66).

In the ADHD sample, we found similar significant and independent effects of the COVID-19 child impact and sleep problems on child mental health for Model I and Model II [β 1.57 (1.22; 1.91) and β 1.91 (1.46; 2.37), respectively]. Similar to the sample without ADHD, we also observed in the ADHD sample a slight reduction in the effect of COVID-19 child impact and sleep problems on the child’s mental health when both variables were included in the same model (Model III) [β 1.25 (0.92; 1.57) and β 1.45 (1.02; 1.88), respectively], and Model III had the lowest AIC values and, as such, was the best model.

Across all tested models, it was found that the effect of COVID-19 child impact within each model was not statistically different between samples without ADHD and ADHD samples as indicated by overlapping confidence intervals. However, sleep problems were found to play a proportionally larger role in children without ADHD when compared to ADHD children [β 3.60 (2.93; 4.27), β 1.91 (1.46; 2.37), respectively, for Model II, and [β 2.72 (1.95; 3.48), β 1.45 (1.02; 1.88)], respectively, for Model III.

### 3.3. The Role of Sleep in Mediating the Relationship of COVID-19 Child Impact on Child Mental Health

**Figure 1:**
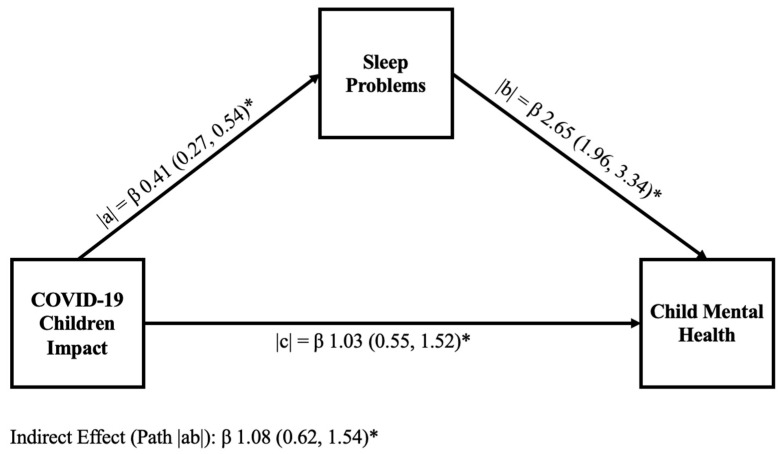
Graphical representation of a mediation model examining the relationship between COVID-19 child impact and child mental health in children without ADHD. The mediation model contains unstandardized coefficients for paths |a|, |b|, the indirect path |ab|, and the direct path |c|, and highlights the mediational role of sleep problems (sleep quality) in the relationship between COVID-19 child impact and child mental health. The direct path represents 48.9%, and the indirect path represents 51.1% of the total mediation. *p* < 0.001 for all paths is represented with *.

**Figure 2:**
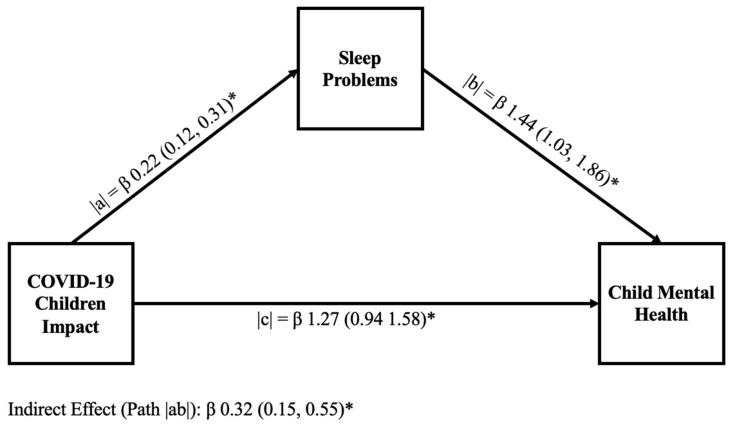
Graphical representation of a mediation model examining the relationship between COVID-19 child impact and child mental health in ADHD children. The mediation model contains unstandardized coefficients for paths |a|, |b|, the indirect path |ab|, and the direct path |c|, and highlights the mediational role of sleep problems (sleep quality) in the relationship between COVID-19 child impact and child mental health. The direct path represents 80%, and the indirect path represents 20% of the total mediation. *p* < 0.001 for all paths is represented with *.

Figure 1 and Figure 2 display the results of mediation analysis models to explain the hypothesized link of sleep problems in mediating the relationship between COVID-19 child impact and child mental health in the samples without ADHD and with ADHD, respectively. In both the without ADHD and with ADHD samples, we identified a significant positive relationship between COVID-19 child impact and the child’s sleep problems (path |a|). We also found a significant positive relationship between the unique effect of sleep problems on child mental health (path |b|). In both samples, we found that sleep problems partially mediate the relationship between COVID-19 child impact and child mental health (path |ab|); however, the values of the estimates were different across samples (children without ADHD [β 1.08 (0.62; 1.54)] and ADHD children [β 0.32 (0.15; 0.55)]). Similarly, the percentage of the mediation described by sleep problems also varied across samples, accounting for 51.1% of the mediation in children without ADHD and 20% in ADHD children.

## 4. Discussion

In this study, we sought to investigate whether sleep plays a role in mediating COVID-19 pandemic impact on mental health in children with and without ADHD. We found that both COVID-19 stress and sleep played a role in impacting child mental health and further found that sleep had a role mediating the impact of COVID-19 on mental health in children with and without ADHD, although to a greater extent in children without ADHD. These results are generally consistent with our hypotheses, except for a smaller moderating role of sleep in the mental health of children with ADHD compared to children without ADHD. Each of the study hypotheses and outcomes is discussed in the following paragraphs.

We hypothesized that both COVID-19 child impact and sleep problems would have a negative association with child mental health in children without ADHD and children with ADHD. We found that in samples without ADHD and with ADHD, COVID-19 child impact and sleep problems were correlated with worse child mental health symptoms (Table 2 and Table 3). This is consistent with the literature where correlations between poor sleep during the COVID-19 pandemic have been linked to worse mental health symptoms in adolescents [74].

We also hypothesized that children with ADHD would experience worse mental health symptoms compared with their peers. We found that, compared to children without ADHD, children with ADHD experienced significantly more distress due to COVID-19 (*p* < 0.001), more sleep disruption (*p* < 0.002), and worse mental health symptoms (*p* < 0.001). The results from this study align with the current literature, which suggests that children with ADHD are at an increased risk of experiencing negative mental health compared to children without ADHD [75,76].

We further hypothesized that sleep problems would mediate the relationship between COVID-19 child impact and child mental health. Based on our mediation analysis, our hypothesis was confirmed: Sleep problems played a significant role in mental health. Path |ab| in Figure 1 and Figure 2 mediated 51.1% and 20% of the relationship between COVID-19 child impact and child mental health in children without ADHD and those with ADHD, respectively. This finding suggests that sleep problems play an important role in child mental health and in the ability of children to cope with chronic stressors like COVID-19. This finding also aligns with the existing literature [49], which suggests that high-quality sleep can be associated with a protective role against negative mental health symptoms. Good sleep is known to contribute to better mental health symptoms [49] and can play a protective role against stressors [77].

Finally, we hypothesized that sleep problems would have a larger explanatory role in children with ADHD compared to children without ADHD. However, despite worse individual symptoms experienced by children with ADHD (COVID-19 child impact, child mental health, and sleep problems scores), our mediation results did not conform with our hypothesis. We found that, in children with ADHD, sleep only mediated 20% of the relationship between COVID-19 child impact on child mental health, compared to 51% for the sample without ADHD. As such, our results suggest sleep problems may play a more significant role in mediating negative mental health symptoms in children without ADHD.

One potential explanation for these findings is that negative mental health symptoms experienced by children with ADHD were more strongly influenced by lifestyle factors not examined in this study, or by a combination of multiple factors (e.g., physical activity, time spent on screens). As a result, the negative mental health symptoms experienced by children without ADHD might be better explained by impacts on one element of their lifestyle (i.e., sleep), leading to a higher mediation value compared to children with ADHD, who may have been more affected by multiple lifestyle changes (i.e., sleep, decreased physical activity, or increased screen time) [78]. Physical activity, for example, has been shown to play an important role in the mental health of children with ADHD [79], while increased screen time has also been shown to play a negative role in mental health [80] in children with ADHD and in worsening ADHD symptoms [81,82]. Additionally, children with ADHD may already experience problems with sleep, and as a result, changes to sleep patterns during the pandemic may have a less pronounced effect in this group due to pre-existing sleep difficulties [42].

Another possible explanation for these findings is that children with ADHD may experience additional unmeasured factors that influence the pathways linking COVID-19 child impact stressors and mental health. Common comorbidities in children with ADHD, such as anxiety [83,84] or learning difficulties [85,86], could allow stressors to impact mental health more directly and potentially reduce the relative mediation of sleep. Similarly, other lifestyle or contextual factors not examined directly in this study, such as the use of stimulant medications, could change sleep patterns in ways that alter the mediating effect of sleep on the relationship between COVID-19 child impact and mental health.

The key strength of this study is that it highlights the interconnectedness of biological factors (such as ADHD and sleep) and psychosocial factors (such as stress) on child mental health. In this study, we incorporate child mental health, sleep problems, and stress to provide a comprehensive view of child well-being and highlight how children with and without ADHD have different vulnerabilities that need consideration. However, several potential limitations of the current study should be considered when interpreting the results. First, an important limitation to consider in this study is that, due to the nature of the measures used, there is a degree of overlap in questions both between measures and with ADHD symptoms (for example, bedtime routine changes are considered in both ADHD symptoms and COVID-19 questions). This could lead to higher correlations in those with neurodevelopmental disorders and could influence results. Furthermore, there is a degree of overlap between symptoms of ADHD, such as inattention and emotional dysregulation, with mental health symptoms included in the CASI-PM-P measure, which might be further exacerbated by poor sleep. This overlap may be reflected in the mediation analysis and could have resulted in sleep problems having an artificially low impact on the total mediation analysis in children with ADHD.

An additional limitation is that, due to the nature of participant selection for the survey (using open public survey links), self-selection bias may occur, since those with knowledge of ADHD or with an interest in ADHD may be more likely to come across the study on the various recruitment platforms and to participate in the study. In addition, due to the nature of answering the survey, recruited parents may be more aware of changes in their child’s behaviour than parents who are not actively looking for changes in their child’s behaviour. Similarly, parent reports of their child’s sleep could be subject to more bias than other methods of collecting sleep data, such as self-reporting or a wearable sleep monitor. For example, pandemic-related stressors may influence parents’ perspectives on their child’s behaviour and could have potentially inflated their responses to study questions. Parental bias ultimately could have influenced the mediation analysis conducted in this study.

Another limitation is the different sample size of the ADHD group (n = 234) and group without ADHD (n = 70), which may underestimate or overestimate the attribution to the outcome (β coefficient), failing to detect the true effect. For instance, the analysis of the population without ADHD (n = 70) is less likely to detect true effects due to the smaller sample size, compared with the similar analysis of the ADHD sample (n = 234), which had a larger sample size.

Another limitation is that, although the study captures variations in COVID-19 stress in children with and without ADHD across Canada, the results of this study may not be generalizable to all children in Canada as the sample was not representative of all Canadian children with and without ADHD. For example, participants were not recruited in proportion to the estimated distribution of children with and without ADHD in each province. Similarly, the study is limited in its generalizability because the study participants largely comprised married, highly educated, and high-income families. This limits the generalizability in particular with respect to those of lower socioeconomic backgrounds who were likely disproportionately impacted by the COVID-19 pandemic and its restrictions. As such, the current results are most applicable to those with higher socioeconomic status.

An additional limitation to consider is that COVID-19 impact is measured using an adapted scale. Although the scale has face validity and a high internal consistency, its psychometric properties have not been fully established in this context.

Another limitation to consider is that due to the cross-sectional nature of the study, we are unable to establish a causal relationship between COVID-19 stress and negative child mental health symptoms or between sleep problems and resultant child mental health symptoms.

A final limitation to consider in this study is that, due to the nature of the methods we used, we did not investigate differences between younger children and early adolescents/teens in their sleep patterns, coping strategies, or pandemic experiences. We recognize that children at different levels of development, and those with/without ADHD, were impacted differently by the pandemic, have different abilities to cope with stressors [87,88], and have different sleep needs. Understanding the role of developmental stage on the relationship between sleep, COVID-19 impact, and child mental health in children with and without ADHD at different developmental stages is something from which future studies would benefit.

Future studies would also benefit from using a broader, balanced, and more representative participant sample to ensure generalizability to the larger population. Further, incorporating multiple lifestyle factors that impact mental health (e.g., sleep, physical activity, diet, support system, etc.) would be helpful to better understand the role of sleep relative to other lifestyle factors. Future work might also consider investigating additional aspects of sleep health, such as sleep quantity, or the impacts of sleep disorders on mental health, and should study the effect of different sleep interventions within the ADHD population. Future studies would also benefit from investigating how different developmental stages impact the relationship between sleep, stress response, and mental health in children with ADHD. Furthermore, we acknowledge that this study uses author-made measures that have not been fully validated in their compressed form (a modified Sleep Disturbance Scale for Children and COVID-19 Child Impact Scale). Although these condensed measures were necessary given the potentially limited time of parents in the context of the pandemic, future studies may consider replicating this study using more validated measures or using more distinct measures to avoid any overlap between measures.

The clinical significance of this study is that it emphasizes the importance of considering strategies that address the multiple factors influencing a child’s mental health while tailoring these strategies to the individual. Clinicians may consider using tools such as sleep diaries, caregiver reports, or questionnaires to identify sleep problems and could consider implementing interventions like educating patients about healthy sleep practices and cognitive-behavioral therapy strategies for insomnia. We highlight how the effects of lifestyle modification strategies (i.e., improving sleep) may differ between children without ADHD and those with ADHD, indicating the need for both diverse and individualized treatment options. Clinicians should identify each patient’s unique needs and develop comprehensive treatment plans incorporating various lifestyle factors, including sleep, to optimize mental health symptoms.

## 5. Conclusions

Sleep problems were found to be a mediator of poorer mental health in children experiencing distress due to COVID-19 in children with and without ADHD. This result underscores the importance of understanding the role of sleep in studies of child mental health and clinical mental health interventions. However, sleep problems were found to have a larger role in mediating mental health symptoms in children without ADHD, underscoring the nuanced impact of sleep and the need for further research in this area, as well as research that also considers additional lifestyle factors, such as physical activity, that may impact child mental health. Overall, our results highlight the important role that sleep plays in mental health and its role in children who are experiencing significant chronic stressors, such as the COVID-19 pandemic.

## Figures and Tables

**Table 1:** Participant demographics.

Variables	ADHD Diagnosis						Total	*p* Value
	No (N 70)			Yes (N 234)				
**Mean Differences (mean+/−SD) ^#^**								
Child’s Age (years)	9.29 +/− 2.54			9.92 +/− 3.08			9.78 +/− 2.97	0.115
Child Mental Health	28.84 +/− 16.64			38.51 +/− 13.24			35.37 +/− 15.20	<0.001 ***
COVID-19 Child Impact	15.54 +/− 5.34			18.84 +/− 4.30			18.08 +/− 4.76	<0.001 ***
Sleep Problems	11.67 +/− 3.78			13.14 +/− 3.31			12.80 +/− 3.47	0.002 **
**Association (N (%)) ^$^**								
**Child Sex**								
Male	35 (50.0)			142 (60.7)			177 (58.2)	0.112
Female	35 (50.0)			92 (39.3)			127 (41.8)	
**Parent Sex**								
Male	27 (38.6)			93 (39.7)			120 (39.5)	0.860
Female	43 (61.4)			141 (60.3)			184 (60.5)	
**Parent Gender**								
Man	27 (38.6)			92 (39.3)			119 (39.1)	0.911
Woman	43 (61.4)			142 (60.7)			185 (60)	
**Parent Relationship**								
Mother/Stepmother	43 (61.4)			139 (59.4)			182 (59.9)	0.762
Father/Stepfather	27 (38.6)			95 (40.6))			122 (40.1)	
**Education**								
Less than High School	1 (1.4)			1 (0.5)			2 (0.7)	0.735
High School	3 (4.3)			5 (2.1)			8 (2.6)	
Some College	6 (8.6)			20 (8.5)			26 (8.6)	
College/University Completed	41 (58.6)			148 (63.2)			189 (62.2)	
Completed Postgrad or more	19 (27.1))			60 (25.6)			79 (26.0)	
**Marital Status (N (%))**								
Not Married	5 (7.1)			21 (9)			26 (8.6)	0.631
Married	65 (92.9)			213 (91.0)			278 (91.4)	
**Region of Canada (N (%))**								
Atlantic Canada	4 (6.0)			29 (12.5)			33 (11.0)	0.167
Central Canada	21 (46.3)			110 (47.4)			141 (47.2)	
Prairies and Northern Territories	16 (23.9)			60 (25.9)			76 (25.4)	
West Coast	16 (23.9)			33 (14.2)			49 (16.4)	
**Household Income**								
CAD 7000 to CAD 12,999	0 (0.0)			3 (1.3)			3 (1.0)	0.057
CAD 13,000 to CAD 19,999	1 (1.4)			3 (1.3)			4 (1.3)	
CAD 20,000 to CAD 26,999	0 (0.0)			5 (2.1)			5 (1.6)	
CAD 27,000 to CAD 32,999	0 (0.0)			6 (2.6)			6 (2.0)	
CAD 33,000 to CAD 39,999	1 (1.4)			11 (4.7)			12 (3.9)	
CAD 40,000 to CAD 52,999	5 (7.1)			17 (7.3)			22 (7.2)	
CAD 53,000 to CAD 65,999	3 (4.3)			38 (16.2)			41 (13.5)	
CAD 66,000 to CAD 79,999	14 (20.0)			34 (14.5)			48 (15.8)	
CAD 80,000 to CAD 92,999	15 (21.4)			31 (13.2)			46 (15.1)	
CAD 93,000 to CAD 105,999	6 (8.6)			15 (6.4)			21 6.9)	
CAD 106,000 to CAD 118,999	3 (4.3)			21 (9.0)			24 (7.9)	
CAD 119,000 to CAD 132,999	2 (2.9)			14 (6.0)			16 (5.3)	
CAD 133,000 to CAD 145,999	4 (5.7)			8 (3.4)			12 (3.9)	
CAD 146,000 to CAD 198,999	7 (10.0)			6 (2.6)			13 (4.3)	
CAD 199,000 to CAD 264,999	4 (5.7)			10 (4.3)			14 (4.6)	
CAD 265,000 to CAD 332,999	3 (4.3)			3 (1.3)			6 (2.0)	
CAD 333,000 to CAD 399,999	0 (0.0)			1 (0.4)			1 (0.3)	
Greater than CAD 400,000	1 (1.4)			4 (1.7)			5 (1.6)	
Prefer not to answer	1 (1.4)			4 (1.7)			5 (1.6)	
**Primary Language (N (%))**								
English	68 (97.1)			232 (99.1)			300 (98.7)	0.170
Other	2 (2.8))			2 (0.9)			4 (1.3)	
**Correlation ^†^**	1	2	3	1	2	3		
Child Mental Health (1)	-	-	-	-	-	-	-	-
COVID-19 Child Impact (2)	0.678 ***	-	-	0.514 ***	-	-	-	-
Sleep Problems (3)	0.792 ***	0.577 ***	-	0.478 ***	0.284 ***	-	-	-
**Collinearity (VIF)**	3.35	1.87	2.71	1.62	1.36	1.3	-	-

Bold font and grey bars indicate participant demographic classifications. ** *p* < 0.01, *** *p* < 0.001; ^#^ Independent t-test, ^$^ Chi-squared association test, ^†^ Pearson’s r.

**Table 2:** Mixed model analysis of children without ADHD.

Characteristics	Model 0	Model I	Model II	Model III
	β(95% CI)	β(95% CI)	β(95% CI)	β(95% CI)
Individual level				
COVID-19 Child Impact	-	2.11 (1.56, 2.66) ***	-	1.00 (0.48, 1.53) ***
Sleep Problems	-	-	3.60 (2.93, 4.27) ***	2.72 (1.95, 3.48) ***
Measure of Variation				
Variance (SD)	5.66 (2.38)	1.74 (1.32)	0.00 (0.00)	0.00 (0.00)
ICC (%)	1.98	1.12	0.00	0.00
Model Fit Statistics				
AIC	573.72	533.46	509.16	497.66

Notes: Mixed model analysis of children without ADHD examining the relationship of COVID-19 child impact and sleep problems on child mental health. Models look at the ability to predict child mental health symptoms first with each variable individually and then together. Positive β coefficients indicate that an increase in a given variable represents a proportional increase in mental health symptom scores (worse mental health). A better model fit is represented by a lower AIC value. *** indicates a *p*-value < 0.001.

**Table 3:** Mixed model analysis of ADHD children.

Characteristics	Model 0	Model I	Model II	Model III
	β(95% CI)	β(95% CI)	β(95% CI)	β(95% CI)
Individual level				
COVID-19 Child Impact	-	1.57 (1.22, 1.91) ***	-	1.25 (0.92, 1.57) ***
Sleep problems	-	-	1.91 (1.46, 2.37) ***	1.45 (1.02, 1.88) ***
Measure of Variation				
Variance (SD)	1.06 (1.03)	0.26 (0.51)	0.39 (0.63)	0.00 (0.00)
ICC (%)	0.60	0.20	0.029	0.00
Model Fit Statistics				
AIC	1861.18	1793.63	1802.91	1754.51

Notes: Mixed model analysis of children with ADHD examining the relationship of COVID-19 child impact and sleep problems on child mental health. The models look at the ability to predict child mental health symptoms first with each variable individually and then together. Positive β coefficients indicate that an increase in a given variable represents a proportional increase in mental health symptom scores (worse mental health). A better model fit is represented by a lower AIC value. *** indicates a *p*-value < 0.001.

## Data Availability

Anonymized data are available upon request to qualified researchers after proper consideration is given to data ethics and existing ethics protocols.

## Supplementary Materials

The following supporting information can be downloaded at: https://www.mdpi.com/article/10.3390/children13010082/s1, Figure S1: Flow chart depicting participant filtration from collection to final analysis; Table S1: Number participants missing questions within a measure broken down by the number of missing questions; Table S2:: Missing at Random Analysis; Table S3: Demographics of participants removed from the study due to missing data; Table S4: Comparison of children without ADHDs retained and removed due to missing data; Table S5: Comparison of ADHD participants retained and removed due to missing data; Table S6: The Child and Adolescent Symptom Inventory-Progress Monitor Parent Survey Questions; Table S7: The Child and Adolescent Symptom Inventory-Progress Monitor Parent Survey Descriptive Statistics for Children without ADHD; Table S8: The Child and Adolescent Symptom Inventory-Progress Monitor Parent Survey Descriptive Statistics for Children with ADHD; Table S9: COVID-19 Child Impact Scale Survey Questions; Table S10: Disorders Initiating and Maintaining Sleep Survey Questions.
