# Supplementary material for: The Role of Sleep in Mediating Mental Health Symptoms During the COVID-19 Pandemic in Children with and Without ADHD
Source: Children (Basel). 2026 Jan 5;13(1):82. doi: 10.3390/children13010082 (PMC12839775; doi:10.3390/children13010082)
Supplement: Supplementary file 1 [file children-13-00082-s001.zip › Revision_Round_2_Children_Supplementary_Information.pdf]

Supplementary Materials

Supplemental Table S1: Number participants missing questions within a measure broken down by the number of missing questions.

Number of Missing Questions	Neurotypical (n=190)			ADHD (n=278)		
	Child Mental Health (n=29)	Covid 19 impact on Children (n=8)	Sleep Quality (n=5)	Child Mental Health (n=29)	Covid 19 impact on Children (n=8)	Sleep Quality (n=5)
**0**	95	110	102	255	264	267
**1**	10	9	11	18	9	8
**2**	0	1	1	1	1	0
**3**	0	0	0	1	1	0
**4**	0	0	0	0	0	0
**5+**	85	70	76	3	3	3

Supplemental Table S2: Missing at Random Analysis

Variables	N	Mean (+/-SD)	Missing [N (%)]
Child Mental Health (CASI)	444	33.82 (+/-15.54)	29 (6.1)
Covid 19 impact on Children	392	20.04 (+/-5.44)	246 (52)
Sleep Quality (DIMSI)	227	12.84 (+/-3.41)	81 (17.1)

Little's MCAR test: Chi-Square = 59.145, Sig. = <0.001

Supplemental Table S3: Demographics of participants removed from the study due to missing data

Variables	ADHD Diagnosis		Total	p value
	No (N 120)	Yes (N 44)		
**Mean Differences (mean+/-SD)** #				
Age (years)	9.60 +/- 2.64	9.68 +/- 2.89	9.58 +/- 2.67	0.875
Child Mental Health	25.25 +/- 12.31	43.44 +/- 15.48	30.46 +/- 15.81	< 0.001***
COVID-19 Child Impact	17.93 +/- 4.37	19.73 +/- 4,56	18.63 +/- 4.47	< 0.097
Sleep Disturbance	12.82 +/- 3.06	13.07 +/- 3.49	12.99 +/- 3.22	0.721
**Association (N (%))** $				
**Child Sex**				
Male	65 (54.2)	25 (56.8)	90 (54.9)	0.762
Female	55 (45.8)	19 (43.2)	74 (45.1)	
**Parent Sex**				
Male	46 (38.3)	15 (34.9)	61 (37.4)	0.688
Female	74 (61.7)	28 (65.1)	102 (62.6)	
**Parent Gender**				
Man	45 (37.5)	12 (27.9)	57 (35.0)	0.258
Woman	75 (62.5)	31 (72.1)	102 (65.0)	
**Relationship**				
Mother/Stepmother	73 (60.8)	28 (63.6)	101 (61.6)	0.802
Father/Stepfather	46 (38.3)	16 (36.4)	62 (37.8)	
Other	1 (0.8)	0 (0.0)	1 (0.6)	
**Education**				
Less than High School	0 (0.0)	0 (0.0)	0 (0.0)	0.394
High School	14 (11.7)	3 (6.8)	17 (10.4)	
Some College	14 (11.7)	2 (4.5)	16 (9.8)	
College/University Completed	71 (59.2)	30 (68.2)	101 (61.6)	
Completed Postgrad or more	21 (17.5)	9 (20.5)	30 (18.3)	
**Marital Status (N (%))**				
Not Married	5 (4.2)	3 (6.8)	8 (4.9)	0.492
Married	114 (95.8)	41 (93.2)	155 (95.1)	
**Province/ Territory (N (%))**				
Atlantic Canada	2 (1.7)	4 (9.1)	6 (3.7)	0.043
Central Canada	69 (57.5)	17 (38.6)	86 (52.4)	
Prairies and Northern Territories	30 (25.0)	13 (29.5)	43 (26.2)	
West Coast	19 (15.8)	10 (22.7)	29 (17.7)	
**Household Income**				
Less than $66,000	23 (19.7)	14 (32.6)	37 (23.1)	0.209

$66,000 - $132,999	81 (69.2)	24 (55.8)	105 (65.6)	
$133,000 or more	13 (11.1)	5 (11.6)	18 (11.8)	
**Primary Language (N (%))**				
English	120 (100)	43 (97.7)	163 (99.4)	0.098
Other	0 (0.0)	1 (2.3)	1 (0.6)	

* p < .05, ** p < .01, *** p < .001

[#] Independent t-test, [$] Chi-squared association test, [†] Pearson's r

Supplemental Table S4: Comparison of children without ADHDs retained and removed due to missing data.

Variables	Neurotypical		Total	p value
	Retained (N 70)	Removed (N 120)		
**Mean Differences (mean+/-SD)** #				
Age (years)	9.29 +/- 2.54	9.60 +/- 2.64	9.48 +/- 2.60	0.424
Child Mental Health	24.84 +/- 16.64	25.25 +/- 12.31	25.08 +/- 14.26	0.856
COVID-19 Child Impact	15.54 +/- 5.34	17.93 +/- 4.37	16.41 +/- 5.12	0.018
Sleep Disturbance	11.67 +/- 3.78	12.82 +/- 3.06	12.11 +/- 3.55	0.093
**Association (N (%))** $				
**Child Sex**				
Male	35 (50.0)	65 (54.2)	100 (52.6)	0.579
Female	35 (50.0)	55 (45.8)	90 (47.4)	
**Parent Sex**				
Male	27 (38.6)	46 (38.3)	73 (38.4)	0.974
Female	43 (61.4)	74 (62.7)	117 (61.6)	
**Parent Gender**				
Man	27 (38.6)	45 (37.5)	72 (37.9)	0.883
Woman	43 (61.4)	75 (62.5)	118 (62.1)	
**Relationship**				
Mother/Stepmother	43 (61.4)	73 (60.8)	116 (61.1)	0.746
Father/Stepfather	27 (38.6)	46 (38.3)	73 (38.4)	
Other	0 (0.0)	0 (0.0)	1 (0.5)	
**Education**				
Less than High School	1 (1.4)	0 (0.0)	1 (0.5)	0.149
High School	3 (4.3)	14 (11.7)	17 (8.9)	
Some College	6 (8.6)	14 (11.7)	20 (10.5)	
College/University Completed	41 (58.6)	71 (59.2)	112 (58.6)	
Completed Postgrad or more	19 (27.1)	21 (17.5)	40 (21.1)	
**Marital Status (N (%))**				
Not Married	5 (7.1)	5 (4.2)	10 (5.3)	0.383
Married	65 (92.9)	114 (95.8)	179 (94.7)	
**Province/ Territory (N (%))**				
Atlantic Canada	4 (6.0)	2 (1.7)	6 (3.2)	0.171
Central Canada	31 (46.3)	69 (57.5)	100 (53.5)	
Prairies and Northern Territories	16 (23.9)	30 (25.0)	46 (24.6)	
West Coast	16 (23.9)	19 (15.8)	35 (18.7)	
**Household Income**				
Less than $66,000	10 (14.5)	23 (19.7)	33 (17.7)	0.016

$66,000 - $132,999	40 (58.0)	81 (69.2)	121 (65.1)	
$133,000 or more	19 (27.5)	13 (11.1)	32 (17.2)	
**Primary Language (N (%))**				
English	68 (97.1)	120 (100)	188 (98.9)	0.177
Other	2 (2.8)	0 (0.0)	2 (1)	

* p < .05, ** p < .01, *** p < .001

Independent t-test, $ Chi-squared association test, † Pearson's r

Supplemental Table S5: Comparison of ADHD participants retained and removed due to missing data.

Variables	ADHD Diagnosis		Total	p value
	Retained (N 234)	Removed (N 44)		
**Mean Differences (mean+/-SD)** #				
Age (years)	9.92 +/- 3.08	9.68 +/- 2.89	9.89 +/- 3.05	0.620
Child Mental Health	38.51 +/- 13.24	43.44 +/- 15.48	39.25 +/- 13.68	0.033
COVID-19 Child Impact	18.84 +/- 4.30	19.73 +/- 4,56	18.94 +/- 4.33	0.287
Sleep Disturbance	13.14 +/- 3.31	13.07 +/- 3.49	13.13 +/- 3.33	0.910
**Association (N (%))** $				
**Child Sex**				
Male	142 (60.7)	25 (56.8)	167 (60.1)	0.631
Female	92 (39.3)	19 (43.2)	111 (39.9)	
**Parent Sex**				
Male	93 (39.7)	15 (34.9)	108 (39.0)	0.548
Female	141 (60.3)	28 (65.1)	169 (61.0)	
**Parent Gender**				
Man	92 (39.3)	12 (27.9)	104 (37.5)	0.156
Woman	142 (60.7)	31 (72.1)	173 (62.5)	
**Relationship**				
Mother/Stepmother	139 (59.4)	28 (63.6)	167 (60.1)	0.599
Father/Stepfather	95 (40.6)	16 (36.4)	111 (39.9)	
Other	0 (0.0)	0 (0.0)	0 (0.0)	
**Education**				
Less than High School	1 (0.4)	0 (0.0)	1 (0.4)	0.367
High School	5 (2.1)	3 (6.8)	8 (2.9)	
Some College	20 (8.5)	2 (4.5)	22 (7.9)	
College/University Completed	148 (63.2)	30 (68.2)	178 (64.0)	
Completed Postgrad or more	60 (25.6)	9 (20.5)	69 (24.8)	
**Marital Status (N (%))**				
Not Married	21 (9.0)	3 (6.8)	24 (8.6)	0.640
Married	213 (91.0)	41 (93.2)	254 (91.4)	
**Province/ Territory (N (%))**				
Atlantic Canada	29 (12.5)	4 (9.1)	33 (12.0)	0.410
Central Canada	110 (47.4)	17 (38.6)	127 (46.0)	
Prairies and Northern Territories	60 (25.9)	13 (29.5)	73 (26.4)	
West Coast	33 (14.2)	10 (22.7)	43 (15.6)	
**Household Income**				
Less than $66,000	83 (36.1)	14 (32.6)	97 (35.5)	0.776

$66,000 - $132,999	115 (50.0)	24 (55.8)	139 (50.9)	
$133,000 or more	32 (13.9)	5 (11.6)	37 (13.6)	
**Primary Language (N (%))**				
English	232 (99.1)	43 (97.7)	275 (98.9)	0.058
Other	2 (0.9)	1 (2.3)	3 (1.1)	

* p < .05, ** p < .01, *** p < .001

Independent t-test, $ Chi-squared association test, † Pearson's r

Supplemental Table S6: The Child and Adolescent Symptom Inventory-Progress Monitor Parent Survey Questions:

Question Prompt: Beside each item below, select the degree of the problem. Consider your child's behaviour in the past month.	Symptom Category	Rating Scale
1. Fails to give close attention to details or makes careless mistakes	Externalizing Symptoms	Never (0) Sometimes (1) Often (2) Very Often (3)
2. Has difficulty paying attention to tasks or play activities		
3. Has difficulty following through on instructions and fails to finish things		
4. Has difficulty organizing tasks and activities		
5. Has difficulty remaining seated when asked to do so		
6. Has difficulty playing/doing things quietly		
7. Is 'on the go' or acts as if 'driven by a motor'		
8. Has difficulty awaiting turn in group activities		
9. Defies or refuses what you tell him/her to do		
10. Is angry and resentful		
11. Takes anger out on others or tries to get even		
12. Does things to deliberately annoy others		
13. Argues with adults		
14. Bullies, threatens, or intimidates others		
15. Starts physical fights		
16. Has deliberately destroyed others' property		
17. Acts restless or edgy	Internalizing Symptoms	
18. Is irritable for most of the day		
19. Is extremely tense or unable to relax		
20. Has difficulty controlling worries		
21. Worries that parents will be hurt or leave home and not come back		
22. Worries that some disaster will separate child from parents		
23. Gets very upset when child expects to be separated from home or parents		
24. Is excessively shy with peers		
25. When put in an uncomfortable social situation, child cries, freezes, or withdraws from interacting		
26. Is depressed/sad for most of the day		
27. Shows little interest (or enjoyment of) pleasurable activities		
28. Has low energy or is tired for no apparent reason		
29. How often do any of the 20 problem behaviours mentioned above interfere with your child's ability to do schoolwork or get along with other people?		

Supplemental Table S7: The Child and Adolescent Symptom Inventory-Progress Monitor Parent Survey Descriptive Statistics for Children without ADHD:

Question Prompt: Beside each item below, select the degree of the problem. Consider your child's behaviour in the past month.	Mean	SD	Rating Scale
1. Fails to give close attention to details or makes careless mistakes	1.343	0.883	Never (0) Sometimes (1) Often (2) Very Often (3)
2. Has difficulty paying attention to tasks or play activities	1.071	0.84	
3. Has difficulty following through on instructions and fails to finish things	0.9	0.801	
4. Has difficulty organizing tasks and activities	1.057	0.849	
5. Has difficulty remaining seated when asked to do so	0.986	0.94	
6. Has difficulty playing/doing things quietly	0.943	0.899	
7. Is 'on the go' or acts as if 'driven by a motor'	0.929	0.953	
8. Has difficulty awaiting turn in group activities	0.871	0.9	
9. Defies or refuses what you tell him/her to do	1.1	0.745	
10. Is angry and resentful	1.157	0.81	
11. Takes anger out on others or tries to get even	0.686	0.894	
12. Does things to deliberately annoy others	0.786	0.832	
13. Argues with adults	0.971	0.884	
14. Bullies, threatens, or intimidates others	0.514	0.794	
15. Starts physical fights	0.643	0.993	
16. Has deliberately destroyed others' property	0.514	0.794	
17. Acts restless or edgy	0.743	0.793	
18. Is irritable for most of the day	0.743	1.017	
19. Is extremely tense or unable to relax	0.786	1.02	
20. Has difficulty controlling worries	0.814	1.011	
21. Worries that parents will be hurt or leave home and not come back	0.829	0.798	
22. Worries that some disaster will separate child from parents	0.986	0.825	
23. Gets very upset when child expects to be separated from home or parents	0.9	0.745	
24. Is excessively shy with peers	0.843	0.895	
25. When put in an uncomfortable social situation, child cries, freezes, or withdraws from interacting	0.8	1.016	
26. Is depressed/sad for most of the day	0.671	0.863	
27. Shows little interest (or enjoyment of) pleasurable activities	0.643	0.817	
28. Has low energy or is tired for no apparent reason	0.714	0.95	
29. How often do any of the 20 problem behaviours mentioned above interfere with your child's ability to do schoolwork or get along with other people?	0.9	0.854	

The median value for questions in the CASI-PM-P was 1, and values for all questions ranged from a minimum of 0 to maximum of 3 for children without ADHD.

Supplemental Table S8: The Child and Adolescent Symptom Inventory-Progress Monitor Parent Survey Descriptive Statistics for Children with ADHD:

Question Prompt: Beside each item below, select the degree of the problem. Consider your child's behaviour in the past month.	Mean	SD	Rating Scale
30. Fails to give close attention to details or makes careless mistakes	1.41	0.701	Never (0) Sometimes (1) Often (2) Very Often (3)
31. Has difficulty paying attention to tasks or play activities	1.504	0.782	
32. Has difficulty following through on instructions and fails to finish things	1.44	0.796	
33. Has difficulty organizing tasks and activities	1.487	0.76	
34. Has difficulty remaining seated when asked to do so	1.444	0.758	
35. Has difficulty playing/doing things quietly	1.521	0.765	
36. Is 'on the go' or acts as if 'driven by a motor'	1.406	0.765	
37. Has difficulty awaiting turn in group activities	1.509	0.737	
38. Defies or refuses what you tell him/her to do	1.363	0.73	
39. Is angry and resentful	1.316	0.799	
40. Takes anger out on others or tries to get even	1.282	0.868	
41. Does things to deliberately annoy others	1.188	0.807	
42. Argues with adults	1.192	0.95	
43. Bullies, threatens, or intimidates others	0.991	0.98	
44. Starts physical fights	0.953	0.937	
45. Has deliberately destroyed others' property	1.124	0.934	
46. Acts restless or edgy	1.385	0.817	
47. Is irritable for most of the day	1.261	0.789	
48. Is extremely tense or unable to relax	1.355	0.812	
49. Has difficulty controlling worries	1.355	0.768	
50. Worries that parents will be hurt or leave home and not come back	1.423	0.857	
51. Worries that some disaster will separate child from parents	1.346	0.841	
52. Gets very upset when child expects to be separated from home or parents	1.432	0.785	
53. Is excessively shy with peers	1.226	0.821	
54. When put in an uncomfortable social situation, child cries, freezes, or withdraws from interacting	1.342	0.755	
55. Is depressed/sad for most of the day	1.244	0.8	
56. Shows little interest (or enjoyment of) pleasurable activities	1.252	0.879	
57. Has low energy or is tired for no apparent reason	1.346	0.805	
58. How often do any of the 20 problem behaviours mentioned above interfere with your child's ability to do schoolwork or get along with other people?	1.415	0.624	

The median value for questions in the CASI-PM-P was 1, and values for all questions ranged from a minimum of 0 to maximum of 3 for children with ADHD.

Supplemental Table S9: COVID-19 Child Impact Scale Survey Questions

Over the last week, has your child experienced changes in any of the following due to COVID-19?	Rating Scale
1. Poorer diet<br>2. Less exercise/physical activity<br>3. Fewer daytime routines and structure<br>4. Less of a bedtime routine<br>5. Increased screen time (e.g., use of phones, tablets, computers, TV, etc.)<br>6. Increased stress<br>7. Increased anxiety<br>8. Decreased socialization	Not at all - 1 (1)<br>A bit - 2 (2)<br>A lot - 3 (3)<br>Completely - 4 (4)

Supplemental Table S10: Disorders Initiating and Maintaining Sleep Survey Questions

**Question Prompt: This questionnaire will allow the research team to have a better understanding of the sleep-wake rhythm.**	**Rating Scale**
1. How long after going to bed does your child usually fall asleep?	Less than (<) 15 minutes (1)<br>15-30 minutes (2)<br>30-45 minutes (3)<br>45-60 minutes (4)<br>More than (>) 60 minutes (5)
**Question Prompt: This questionnaire will allow the research team to have a better understanding of the sleep-wake rhythm of your child and of any problems in their sleep behaviour. When answering these questions please consider what your child's sleep behaviour is usually like over the past month.**	**Rating Scale**
2. The child goes to bed reluctantly<br>3. The child has difficulty getting to sleep at night (and may require a parent to be present)<br>4. The child wakes up two or more times a night<br>5. After waking up in the night the child has difficulty falling asleep again by himself or herself	The behaviour never occurs (1)<br>The behaviour occurs 1 or 2 times a month (2)<br>The behaviour occurs 1 or 2 times a week (3)<br>The behaviour occurs between 3 and 5 nights a week (4)<br>The behaviour happens every night (5)

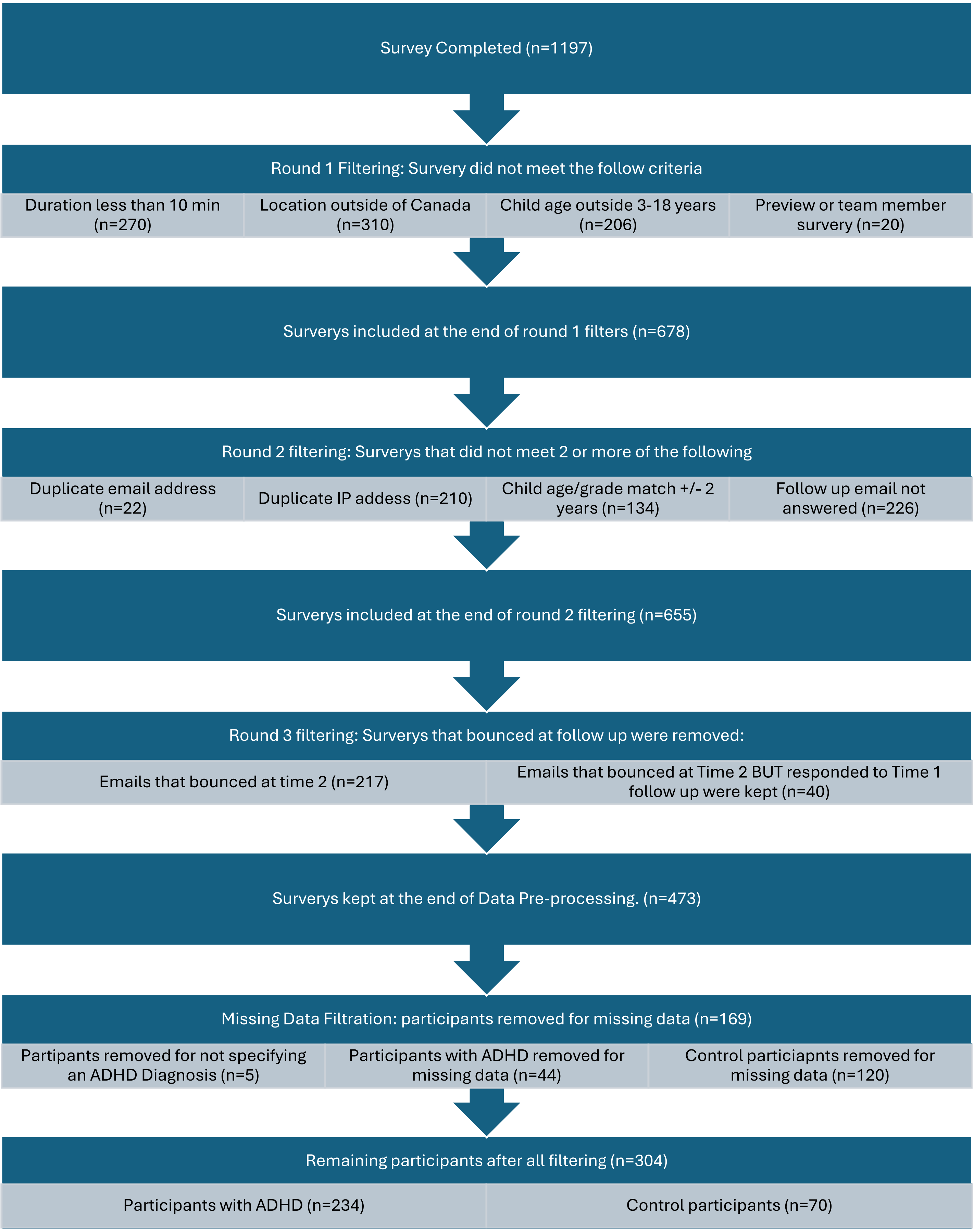

Supplemental Figure S1: Flow chart depicting participant filtration from collection to final analysis.
